# The Prevalence and Level of Awareness of Medication Overuse Headache in Qassim Province, Saudi Arabia: A Cross-Sectional Study

**DOI:** 10.7759/cureus.28101

**Published:** 2022-08-17

**Authors:** Samer A Almuqairsha, Mohammad I Aldekhail, Abdullah I Aldekhail, Mohammed H Alresaini, Sulaiman S Almarshoud, Salman A Alashqar, Ibrahim Algosair, Haitham H Alresaini

**Affiliations:** 1 Department of Internal Medicine, Qassim University, Buraydah, SAU; 2 College of Medicine, Qassim University, Buraydah, SAU

**Keywords:** qassim province, prevalence, awareness, chronic headache, medication overuse headache

## Abstract

Introduction

Medication overuse headache (MOH) is a secondary headache caused by regular overuse of medication(s) intended to relieve (notably, not prevent) the symptoms of a pre-existing primary headache (most commonly, migraine and tension headaches). MOH places a considerable burden on both patients and society. Understandably, the disorder is the subject of many cross-sectional studies worldwide. Research on MOH in Qassim province, Saudi Arabia, however, has not been conducted; therefore, the present study aims to identify the prevalence and level of awareness of MOH in Qassim province.

Methods

An observational cross-sectional study was conducted in Qassim province from July 1, 2021, to June 13, 2022. A modified electronic questionnaire was distributed through social media platforms. The questionnaire covers demographics, prevalence of MOH, and awareness of MOH. Prevalence was measured using a scoring system based on the criteria of the International Classification of Headache Disorders, third edition (ICHD-3) for diagnosing MOH.

Results

A total of 499 people completed the questionnaire, 286 (57.3%) of whom were female and 213 (42.7%) of whom were male. The majority of the participants were Saudi nationals (95.4%). The mean age of the participants was 35 years old (± 12.7 years). Out of the 499 participants, 451 (90.4%) reported that they had experienced headaches in their life. The prevalence of MOH among those who had reported headaches was 4%, compared with a level of awareness of MOH among all the participants of 18%.

Conclusion

According to our study, there is a high prevalence of MOH in Qassim province. This is also coupled with a high awareness of MOH.

## Introduction

Medication overuse headache (MOH), also known as medication-misuse headache and rebound headache, is a secondary headache caused by regular overuse of medication(s) intended to relieve (notably, not prevent) the symptoms of headaches [1]. The conditions of most MOH patients improve after the cessation of the overused medication [1]. According to the International Classification of Headache Disorders, third edition (ICHD-3), the diagnostic criteria for MOH are (A) headache occurring on ≥15 days/month in a patient with a preexisting primary headache, (B) regular overuse of one or more drugs for acute or symptomatic headache treatment for >three months, and (C) symptoms that cannot be better accounted for by another ICHD-3 diagnosis [1]. The definition of regular overuse depends on the type of medication. Overuse is considered to be the use for ≥15 days/month of simple analgesics (e.g., paracetamol and non-steroidal anti-inflammatory drugs (NSAIDs)) and ≥10 days/month of opioids, triptans, and combination analgesics [1].

In primary care, most MOH cases are caused by over-the-counter (OTC) medications, compared to secondary and tertiary care, where most cases are caused by centrally acting and more potent medications. The most common MOH-causing medications have differed over time and across regions. Unlike in the past, MOH caused by ergotamine overuse is no longer a common issue in Western Europe. In its place, triptans are now one of the most common medications that cause MOH in the Western world [2].

As one of the most common chronic headache disorders, affecting around 63 million people worldwide, MOH’s prevalence varies globally, with a range of 0.5-7.2% across countries, likely due to different definitions of regular medication overuse [3-6]. The prevalence of probable MOH (pMOH), which is defined as headaches on ≥15 days/month associated with medication overuse, is highest in Russia, with a prevalence of 7.2% [7]. In the United States, 23.3% of chronic headache patients use acute medication every day, compared to only 9% in Norway [8,9]. Despite limited data available in developing countries, recent studies have found the prevalence in Africa (Zambia, 7.1%; Ethiopia, 0.7%), Latin America (Brazil, 1.4%; Colombia, 4.3%), and Asia (Korea, 0.5%; China, 0.6%) [10]. In addition to geographic variation, there is also demographic variation. MOH affects women more than men, with a male-to-female ratio of 1:3.5 [11]; this variation could be explained by the fact that headache disorders are more common among women, thus making the possibility of overusing headache medication higher among women. As for age, MOH usually develops in the fourth decade of life, after which the prevalence decreases with age. In Taiwan, for example, the prevalence among people over 65 years was 1.0% and estimated to be 0.3-0.5% among children and adolescents [4,12].

Patients with headache disorders usually prefer self-medication, which can be problematic when these medications are overused, eventually resulting in MOH, especially considering that most MOH-causing medications are easy-to-access analgesics [1,13,14]. As an unappreciated global health issue, MOH is also the costliest headache in Europe, with an annual cost estimated at €3,561 per person [15]. In addition to its economic impacts, MOH represents a physical and social burden. A study found that 44.7% of men and 53.7% of women with pMOH lose >20 workdays every three months, which is double the days lost due to migraine [16]. According to The Global Burden of Diseases, Injuries, and Risk Factors Study 2016 (GBD 2016), migraine (which includes MOH as a sequel of migraine) is the second largest cause of disability, which represents an upward trend, with migraine placing sixth in GBD 2013 and third when combined with MOH [5,17]. Fortunately, the burden of MOH can easily be avoided, given that it is a preventable and treatable disorder.

The prevention and treatment of MOH, however, requires awareness of the disorder, which is not widely known. A cross-sectional study conducted on 485 participants at the University of Birmingham in the United Kingdom found that 77% of the participants were unaware of the possibility of MOH resulting from regular analgesic use for headaches. After education on MOH, 80% of them stated that they would decrease analgesic use or seek medical advice [18]. Moreover, the level of awareness can be increased through public campaigns; in Denmark, for example, the Danish Headache Center, the Association of Danish Pharmacies, and headache patient organizations carried out a four-month MOH awareness campaign in 2016, which increased the Danish adult population’s awareness from 31% to 38% [19].

In Saudi Arabia, MOH has received only limited academic attention, affirming the importance of conducting more research on this subject. In addition, the common use of analgesics in the country is not accompanied by sufficient awareness of their adverse effects. In a cross-sectional study performed in Riyadh, the capital of Saudi Arabia, on paracetamol’s possible side effects, 87.6% of the 323 respondents were unaware that paracetamol could cause chronic headaches [20]. In another study at the national level, 2% of the respondents had pMOH, with a female predominance, as well as those aged between 46-55 years being the most prevalent age group [21]. Since the prevalence and level of awareness of MOH in Qassim province have not been studied before, the present study aims to fill this research gap.

## Materials and methods

This observational cross-sectional study was conducted at Qassim University, Buraydah, Saudi Arabia, from July 1, 2021, to June 13, 2022. A modified electronic questionnaire was distributed through social media platforms while ensuring that only those who live in Qassim province were included. The questionnaire was based on the ICHD-3 criteria for diagnosing MOH and has been used in previous research conducted in Riyadh [20]. The questionnaire contained 10 questions, starting with three multiple-choice questions and one open-ended question to collect demographic data (i.e., gender, age, nationality, and educational level), followed by two multiple-choice questions on whether the participants have ever had a headache in the past and the number of days they experienced headaches within the last 30 days; one checkbox question on the medication(s) that the participants use to relieve their headaches; two multiple-choice questions on the number of days on which the participants used headache medications within the last 30 days and how long the participants usually continue using headache medications; and finally, one checkbox question on the side effects of headache medications to assess whether the participant is aware of MOH. This study uses a scoring system for the diagnosis of MOH based on the participant’s answers to four questions. The four questions have a total score of 11 points; if a participant’s answers added up to eight or more points, they were considered to have MOH (Table 1).

**Table 1:** Scoring system for diagnosing MOH MOH: medication overuse headache

Question	No points	One point	Two points	Three points	Four points
For how many days have you experienced headaches within the last 30 days?	Did not experience any headaches	1-2 days	3-7 days	8-14 days	15 days or more
Which of the following medications do you use to relieve your headache?	Does not use medications	Use medications			
For how many days have you used these medications to relieve your headache within the last 30 days?	Did not use headache medications within the last 30 days	1-9 days	10-14 days	15-30 days	
For how long do you usually use these medications to relieve your headache?	Intermittently	Less than a month	1-3 months	More than 3 months	

The first question in our scoring system was, “For how many days have you experienced headaches within the last 30 days?” The answer “I did not experience any headaches within the last 30 days” received no points, “one to two days” one point, “three to seven days” two points, “eight to 14 days” three points, and “15 days or more” four points. The second question was, “Which of the following medications do you use to relieve your headache?” This question was only worth one point, which was added to the scores of participants who used headache medications, namely paracetamol, NSAIDs, Solpadeine, and/or sumatriptan. The third question was, “For how many days have you used these medications to relieve your headache within the last 30 days?” The answer “Did not use headache medications within the last 30 days” received no points, “one to nine days” one point, “10 to 14 days” two points, and “15 to 30 days” three points. The fourth and final question in our scoring system was, “For how long do you usually use these medications to relieve your headache?” The answer “intermittently” received no points, “less than one month” one point, “one to three months” two points, and “more than three months” three points. Any participant with eight or more points in total was considered to have MOH. In addition, the questionnaire measured the awareness of MOH by asking about the side effects of the abovementioned medications in a checkbox question that contained multiple choices (one of which was chronic headache). Those who chose chronic headache as a side effect were considered to be aware of MOH.

Statistical analysis of the recorded data was performed using the IBM SPSS Statistics for Windows, Version 24.0 (Released 2016; IBM Corp., Armonk, New York, United States). The categorical data was recorded as frequency and percentages, and the quantitative data was recorded as mean and standard deviation. This study obtained ethical approval from the Committee of Research Ethics, Deanship of Scientific Research, Qassim University (Number 21-05-10).

## Results

A total of 499 people completed the questionnaire, 286 (57.3%) of whom were women and 213 (42.7%) of whom were men. The majority of the participants were Saudi nationals (95.4%). Most of the participants held a bachelor’s degree (64.9%), followed by high school degrees as the second most common highest degree held by the participants (24%). The mean age of the participants was 35 years old (± 12.7 years) (Table 2).

**Table 2:** Demographic characteristics of the participants (n=499)

Variable		Frequency	Percentage
Gender	Female	286	57.3%
	Male	213	42.7%
Nationality	Saudi	476	95.4%
	Non-Saudi	23	4.6%
Education level	Less than high school	18	3.6%
	High school	120	24.0%
	Bachelor's degree	324	64.9%
	Master's degree	27	5.4%
	PhD degree	10	2.0%
Age	Mean	35.38 years old	
	Standard deviation	+-12.7 years	

Out of the 499 participants, 451 (90.4%) reported that they had experienced headaches in their life. Regarding the frequency of headaches experienced within the last 30 days, 226 (50.1%) had experienced one or two days with a headache, 127 (28.2%) three to seven days with a headache, 31 (6.9%) eight to 14 days with a headache, and 23 (5.1%) more than 15 days with a headache. The remaining 44 (9.8%) participants had not experienced any headaches within the last 30 days (Table 3).

**Table 3:** Participants' headache history

Question	Choices	Frequency	Percentage
Have you ever had a headache in the past?	No	48	9.6%
	Yes	451	90.4%
Total		499	100%
For how many days have you experienced headaches within the last 30 days?	I did not experience any headaches within the last 30 days	44	9.8%
	1 - 2 days	226	50.1%
	3 - 7 days	127	28.2%
	8 - 14 days	31	6.9%
	15 days or more	23	5.1%
Total		451	100%

The majority of the participants, 302 (67%), chose paracetamol to relieve their headaches. The second most common medication was NSAIDs, as the preferred choice of 51 (11.3%) participants. Solpadeine and sumatriptan were the choices of 24 (5.3%) and eight (1.8%) participants, respectively. Almost a third of the participants, 133 (29.5%), stated that they do not use any kind of medication to relieve their headaches (Table 4).

**Table 4:** Types of headache medication(s) used by the participants NSAIDs: non-steroidal anti-inflammatory drugs

Question	Choices (Participants can choose more than one).	Frequency	Percentage
Which of the following medications do you use to relieve your headache?	I don’t use medications for headache	133	29.5%
	Paracetamol (Panadol, Fevadol, Adol, Panadrex, or Acitam)	302	67%
	NSAIDs (aspirin, ibuprofen, or diclofenac)	51	11.3%
	Solpadeine	24	5.3%
	Sumatriptan	8	1.8%

Of those who had experienced headaches, 180 (39.9%) had not taken any medication to relieve their headaches within the last 30 days. This is compared to 227 (50.3%) who used medications for one to nine days, 29 (6.4%) for 10 to 14 days, and 15 (3.3%) for more than 15 days. Regarding the duration of medication use, 342 (75.8%) of the participants use medications intermittently, 67 (14.9%) for less than one month, 10 (2.2%) for one to three months, and 32 (7.1%) for more than three months (Table 5).

**Table 5:** Participants' history of headache medication(s)

Question	Choices	Frequency	Percentage
For how many days have you used these medications to relieve your headache within the last 30 days?	Did not use headache medications within the last 30 days	180	39.9%
	1-9 days	227	50.3%
	10-14 days	29	6.4%
	15-30 days	15	3.3%
For how long do you usually use these medications to relieve your headache?	Intermittently	342	75.8%
	Less than one month	67	14.9%
	1-3 months	10	2.2%
	More than three months	32	7.1%

Based on the scoring system, the prevalence of MOH is 4% of the 451 participants who reported that they had experienced headaches in their life (Table 6).

**Table 6:** The results of the scoring system

Score	Frequency	Percentage
0	13	2.9%
1	81	18.0%
2	58	12.9%
3	109	24.2%
4	93	20.6%
5	39	8.6%
6	17	3.8%
7	23	5.1%
8	9	2.0%
9	4	0.9%
10	4	0.9%
11	1	0.2%
Total	451	100%

Regarding MOH awareness, 90 (18%) of the participants chose chronic headaches as a side effect of headache medications and were thus considered to be aware of MOH (Table 7).

**Table 7:** Headache medications' side effects

Side effects (Participants can choose more than one)	Frequency	Percentage
Skin rash	28	5.6%
Chronic headache	90	18%
Tiredness or weakness	178	35.7%
Loss of appetite	121	24.2%
Diarrhea	77	15.4%
Fever	40	8%
Increased sweating	114	22.8%
Stomach cramps	184	36.9%
Bloody urine	8	1.6%

## Discussion

MOH is a secondary headache caused by regular overuse of medication(s) intended to relieve (notably, not prevent) the symptoms of a pre-existing primary headache. [1]. MOH is one of the most common chronic headache disorders affecting around 63 million people worldwide [3-5]. Two studies, one conducted at the University of Birmingham in the United Kingdom and the other in Riyadh, show that 77% and 87.6% of the participants, respectively, were unaware of MOH [18,20]. Considering MOH burden on patients and society and the high rate of MOH unawareness worldwide and locally, we wanted to identify the prevalence and level of awareness of MOH in Qassim province.

Data was gathered by distributing an online questionnaire on social media platforms. Accordingly, there is a potential responder bias, as not everyone in the targeted population has access to social media. Regarding MOH awareness, out of the 499 people who completed the questionnaire, 82% of them were unaware of MOH. While this is high, Qassim province has a relatively lower unawareness rate compared to the results from the abovementioned study in Riyadh, which found that 283 (87.6%) of its participants were unaware of MOH [20]. The mean age of the participants in the current study was 35 years old (±12.7 years), which is an expected outcome, as this age group constitutes the majority of social media users [22].

In this study, we found that MOH is more common among women, which is likely due to the fact that migraine (a predisposing factor of MOH) is more common among women [23,24]. Moreover, over half (57.3%) of the participants in the current study were women, which could be because of the high prevalence of MOH among females, i.e., they may have been interested in participating and completing the questionnaire. Most (64.9%) of the participants had a bachelor’s degree as their highest degree, which does not reflect the general population [25]. Considering that the education level of our participants is higher than the general population, the level of awareness of MOH among the general population might be lower than what is noted in this study.

In our sample study, paracetamol is the most commonly used drug, i.e., used by 302 (67%) participants, owing to the fact that it is presumed to be safe (with minimal side effects) compared to other analgesics. [26,27].

Regarding the prevalence of MOH, 451 (90.4%) of the participants reported that they had experienced headaches in their life, but only 18 (4%) fulfilled the criteria for MOH. For comparison, at the national level and also in Denmark, the prevalence of MOH is only 2% [21,28].

## Conclusions

The prevalence of MOH in Qassim province is high. This is also coupled with a high awareness of MOH; this could be explainable by the high educational level of our sample. More studies are needed to measure the local prevalence and awareness of MOH while addressing factors like co-morbidities, patient's weight, stress, and diet. Health awareness programs targeting the community at a local level are essential given the high prevalence of MOH.

## Appendices

Questionnaire

Demographics

1. Gender:

A/ Male                                       B/ Female

2. Nationality:

A/ Saudi                                       B/ non-Saudi

3. What is your educational level:

A/ Less than High school.

B/ High school.

C/ Bachelor’s degree.

D/ Master’s degree.

F/ Ph.D.

4. Age: ………Years.

Headache

5. Have you ever experienced a headache in the past?

A/ Yes                                               B/ No

(If you answered No, skip to the last question)

6. For how many days have you experienced headaches within the last 30 days?

A/ Did not experience any headaches

B/ 1-2 days

C/ 3-7 days

D/ 8-14 days

F/ 15 days or more

Medication

7. Which of the following medications do you use to relieve your headache?

A/ I don’t use medications for headache

B/ Paracetamol (Panadol, Fevadol, Adol, Panadrex, or Acitam)

C/ NSAIDs (aspirin, ibuprofen, or diclofenac)

D/ Solpadeine

F/ Sumatriptan

8. For how many days have you used these medications to relieve your headache within the last 30 days?

A/ Did not use headache medications within the last 30 days

B/ 1-9 days

C/ 10-14 days

D/ 15-30 days

9. For how long do you usually use these medications to relieve your headache?

A/ Intermittently

B/ Less than one month

C/ 1-3 months

D/ More than three months

10. Please select all the headache medication side effects that you are aware of (more than one answer can be selected).

● Diarrhea

● Increased sweating

● Loss of appetite

● Stomach cramps

● Skin rash

● Chronic headache

● Fever

● Bloody urine

● Tiredness or weakness
